# *Bacillus thuringiensis* Spores and Cry3A Toxins Act Synergistically to Expedite Colorado Potato Beetle Mortality

**DOI:** 10.3390/toxins13110746

**Published:** 2021-10-21

**Authors:** Ivan M. Dubovskiy, Ekaterina V. Grizanova, Daria Tereshchenko, Tatiana I. Krytsyna, Tatyana Alikina, Galina Kalmykova, Marsel Kabilov, Christopher J. Coates

**Affiliations:** 1 Department of Plant Protection, Novosibirsk State Agrarian University, 630039 Novosibirsk, Russia; katalasa_2006@yahoo.com (E.V.G.); tereshenko-darya@mail.ru (D.T.); krytsyna@list.ru (T.I.K.); 2 Siberian Federal Scientific Centre of Agro-BioTechnologies, Russian Academy of Sciences, 630501 Krasnoobsk, Russia; gvkalmyk@mail.ru; 3 Institute of Chemical Biology and Fundamental Medicine, Siberian Branch of the Russian Academy of Sciences, 630090 Novosibirsk, Russia; alikina@niboch.nsc.ru (T.A.); kabilov@niboch.nsc.ru (M.K.); 4 Department of Biosciences, Faculty of Science and Engineering, Swansea University, Swansea SA2 8PP, Wales, UK; C.J.Coates@swansea.ac.uk

**Keywords:** immunotoxicology, ROS, oxidative stress, antioxidants, midgut microbiome, biocontrol, *Leptinotarsa decemlineata*, plant protection

## Abstract

The insect integument (exoskeleton) is an effective physiochemical barrier that limits disease-causing agents to a few portals of entry, including the gastrointestinal and reproductive tracts. The bacterial biopesticide *Bacillus thuringiensis* (Bt) enters the insect host via the mouth and must thwart gut-based defences to make its way into the body cavity (haemocoel) and establish infection. We sought to uncover the main antibacterial defences of the midgut and the pathophysiological features of Bt in a notable insect pest, the Colorado potato beetle *Leptinotarsa decemlineata* (CPB). Exposing the beetles to both Bt spores and their Cry3A toxins (crystalline δ-endotoxins) via oral inoculation led to higher mortality levels when compared to either spores or Cry3A toxins alone. Within 12 h post-exposure, Cry3A toxins caused a 1.5-fold increase in the levels of reactive oxygen species (ROS) and malondialdehyde (lipid peroxidation) within the midgut – key indicators of tissue damage. When Cry3A toxins are combined with spores, gross redox imbalance and ‘oxidation stress’ is apparent in beetle larvae. The insect detoxification system is activated when Bt spores and Cry3A toxins are administered alone or in combination to mitigate toxicosis, in addition to elevated mRNA levels of candidate defence genes (pattern-recognition receptor, stress-regulation, serine proteases, and prosaposin-like protein). The presence of bacterial spores and/or Cry3A toxins coincides with subtle changes in microbial community composition of the midgut, such as decreased *Pseudomonas* abundance at 48 h post inoculation. Both Bt spores and Cry3A toxins have negative impacts on larval health, and when combined, likely cause metabolic derangement, due to multiple tissue targets being compromised.

## 1. Introduction

*Bacillus thuringiensis* (Bt) is a widespread bacterium that has been developed as a biopesticide to control insect pests as well as arbovectors, e.g., mosquitoes [1]. The insecticidal activity of Bt is primarily due to crystalline endotoxins (Cry toxins) produced during sporulation and activated by proteases in the host’s gut [2]. The binding of activated toxins to receptors on the surface of midgut epithelial cell membranes either creates pores that cause cell lysis, or they activate intracellular signalling pathways that result in cell death (i.e., swelling) [3,4].

Cry toxins can kill insects alone (when expressed in genetically modified plants), yet there are many virulence factors deployed by spores and vegetative cells that contribute to bacterial pathogenicity. Some lipids, carbohydrates and enzymes associated with the bacterial exosporium help Bt to adhere to host tissues, germinate and protect the spores from elimination by insect defence systems [5]. Bt-associated virulence factors, which are transcribed in the vegetative cells by the PlcR-PapR quorum-sensing system, play an important role in the infection process [6]. Vegetative *Bt* cells can release vegetative and secreted insecticidal proteins, Vip and Sip, which affect coleopteran larvae, including the Colorado potato beetle *Leptinotarsa decemlineata* [7,8]. Enhancin-like, InhA and other metalloproteases, chitinases, phospholipase, cytolysins and some non-proteinaceous factors (zwittermycin A, β-exotoxins) produced by Bt cells are also linked to virulence and infection outcomes, due to cytotoxic effects or as regulators of major toxin activity [9,10]. There is evidence that bacteria could express some of these virulence and homeostatic factors during insect colonisation in order to overcome host defenses [11,12]. 

The CPB is a dangerous pest in Eurasia, North America and Africa. CPB can propagate and acclimate in a wide range of habitats due to their high plasticity, migration capacity and intraspecific polymorphism [13,14]. Bt is widely used to control CPB in the field via foliar sprays and Bt transgenic plants [15]. However, there are increasing reports of CPB resistance to Bt [16,17,18] that compels us to further investigate Bt-CPB interactions. The mechanisms of insect resistance to Bt have been studied extensively and are multifaceted, especially in the case of Bt spray application. Resistance is likely to be multigenic because bacterial virulence factors, such as the spore, toxins and enzymes can play a vital role in the overall toxicity of Bt-based insecticides.

The defence strategies of insects against Bt are multi-factorial, which are focused in the midgut, as a key antibacterial barrier [19,20,21]. Detection of pathogens with pattern-recognition receptors (C-type lectins, PGRPs, TEPs etc.) and damage-associated molecular patterns in the midgut is important to accelerate local defence reactions [22]. Cathepsins, which are found in lysosomes, are involved in numerous physiological and pathological processes (apoptosis, intracellular protein degradation, and hormone maturation), including the immune response against microbial infection in insects [23]. Active protection of insects against Bt Cry toxins include the sequestration of the toxin by lipophorin, esterases or alkaline phosphatase and increased stem cell production in the gut to replace damaged epithelial cells [24,25,26], as well as the release of antimicrobial peptides [21,27]. When Bt toxins bind to the receptors on epithelial cell surfaces, the integrity of membranes is disrupted, leading to lipid peroxidation, redox imbalance and dysregulated ROS production [21,28]. Thus, ROS accumulation can be one of the co-factors leading to systemic disorders in an insect during Bt toxicosis. Insect cells are able to defend themselves against ROS damage through the usage of chemical and enzymatic antioxidants. Major components of the insect detoxification system include superoxide dismutases, several ascorbate peroxidases, catalases, peroxidases, glutathione-S-transferase (GST), ascorbic acid, thiols and α-tocopherol [29]. In addition, GST, esterases, cytochrome P450 monooxygenases and lysosomal factors (e.g., saposins, cathepsins) are able to remove toxic compounds and the products of lipid peroxidation or hydroperoxides from cells [30,31]. Thiols defend cells from damaging hydroxyl radicals, nitroxyl radicals and superoxide radicals [32]. The oxidation of SH-containing compounds results in a decrease in reduced SH-groups (RSH) and an increase in oxidated SH groups (RSSR). The levels of lipid peroxidation and the thiols ratio (RSSR/RSH) are considered markers of redox balance [33]. Moreover, the insect gut microbiota can interfere with Bt efficacy either by activating/degrading the toxin or initiating/protecting against septicaemia [34,35,36]. Bt dwelling in the host cadaver propagate until they exhaust all available organic materials prior to sporulation [6].

For some insects, enhanced virulence of Bt requires the presence of germinated vegetative bacteria and spores, in addition to Cry toxins [37]. Spores play a crucial role in the pathogenesis of Bt in lepidopteran larvae, such as wax moth (*Galleria mellonella*) [37,38], diamondback moths (*Plutella xylostella*) [39], Indianmeal moth (*Plodia interpunctella*) [40] and western spruce budworm (*Choristoneura occidentalis*) [41]. Some toxins and enzymes brought with Bt spores or produced by vegetative cells could be important in bacterial pathogenesis [42]. Thus, the potentiating effect of spores on crystal (Cry toxins) toxicity has been known for years while the mechanism of the synergistic effect remains unclear [43].

In this paper, we explore the synergistic effect of spores and Cry3A toxins related to the virulence of *Bacillus thuringiensis* ssp. morrisoni var. thuringiensis toward the economically devastating pest Colorado potato beetle *L. decemlineata*. We report on the repertoire of midgut defence reactions of CPB larvae that work to protect insects from Bt virulence factors. The goal is to identify features in the infected insects that could account for their increased susceptibility when treated with a Bt spore–crystal mixture. To this end, we examine antioxidant responses, humoral immunity, stress and toxin management, and changes in the gut microbiome in CPB larvae inoculated with Bt spores, Cry3A toxins and their mixture. 

## 2. Results

### 2.1. Susceptibility of CPB Larvae to Bt Treatments

**Figure 1:**
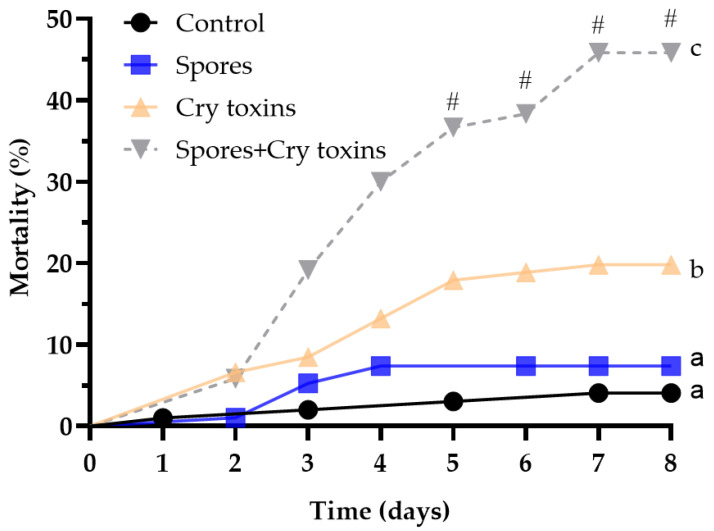
Mortality of Colorado potato beetle larvae following oral treatment with *Bacillus thuringiensis* spores and Cry3A toxins. The control consisted of administering PBS alone (no spores or toxins). Data were analysed by comparing curves using Log-rank (Mantel–Cox) tests (*n* = 100 larvae per treatment). The hashtag (#) denotes differences between treatments (binomial test) at timepoints 5–8. Unshared letters represent significant differences (*p* < 0.05).

Overall, Bt-derived spores and crystal (Cry) endotoxins had significant negative effects on the health of Colorado potato beetle (CPB) larvae (*X*^2^_(3)_ = 74.19, *p* < 0.0001; Figure 1, Table 1). Exposing larvae to the combined spore and Cry toxin inoculum via gavage led to the most severe increase in mortality ~46% over the 8-day experimental period (Figure 1). These insects were 6.3-fold more likely to die when compared to the negative control and between 2.6 and 4.9-fold more likely to die when compared to each Bt-derived treatment (Table 1). Mortality levels reduced by ∼7% and ∼20% when spores and Cry toxins, respectively, were administered individually. Variation in insect death was noticeable from day 3, where levels were twice as high for those receiving the spores/Cry toxin mix, and were significantly different from day 5 onwards. We detected with the binomial test the synergistic effect in CPB mortality for Bt mixture spores with the Cry toxin variant from the fifth day post treatment (Chi square = 7; 9; 11; 11 accordingly). These data suggest that spores and Cry toxins act synergistically to kill insects at much higher levels over a shorter period. Only 4% of control larvae—receiving PBS only—died during the experiment and were not considered significantly different to those receiving spores (Figure 1; Table 1).

### 2.2. Redox Balance and Detoxification in CPB Larvae Post Bt Treatment

**Figure 2:**
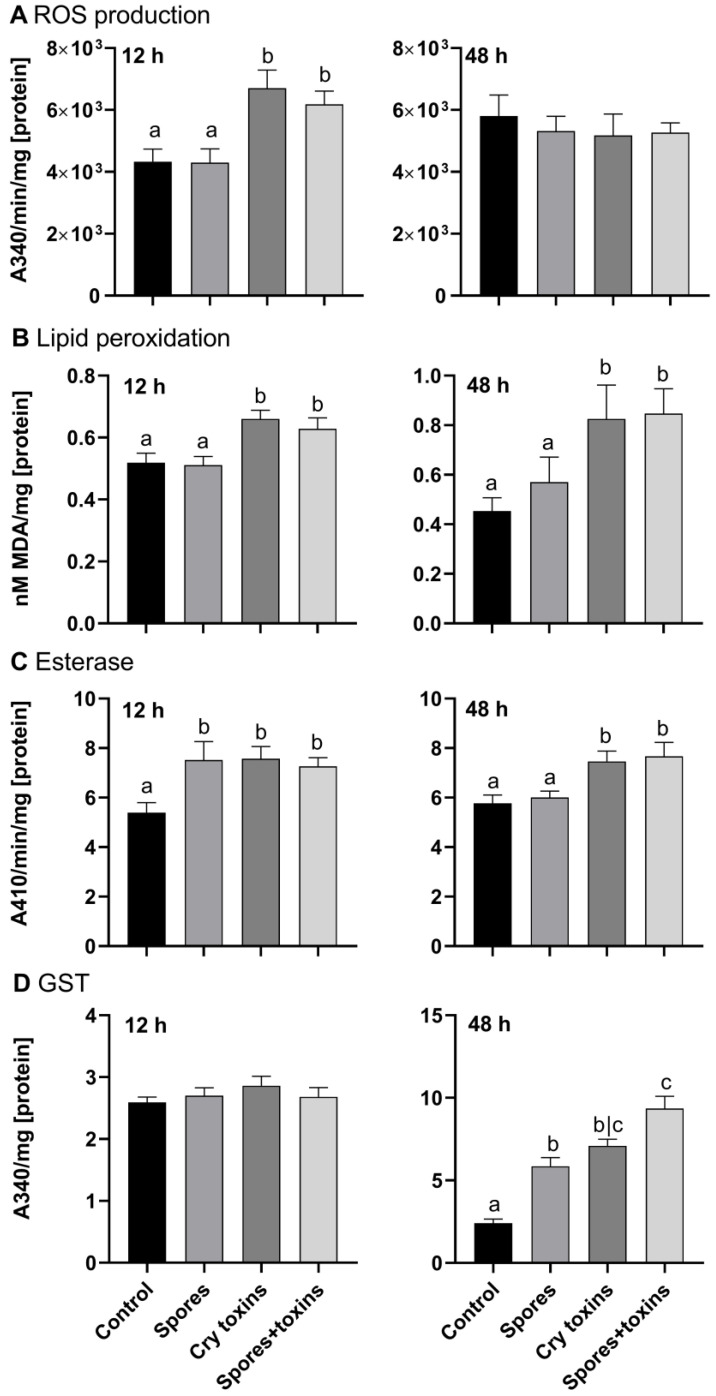
Reactive oxygen species (ROS) generation (**A**), lipid peroxidation (**B**), esterase (**C**) and glutathione-S-transferase (GST) (**D**) activities in the midgut of Colorado potato beetle larvae treated with *Bacillus thuringiensis* spores and Cry3A toxins. The negative control consisted of administering PBS alone (no spores or toxins). Panels in the left column are measures taken at 12 h and panels in the right column are measures taken at 48 h post inoculation. Data represent mean ± SE. Unshared letters represent significant differences (*p* < 0.05), determined via ANOVA or Kruskal–Wallis (with Dunnett’s or Dunn’s multiple comparisons).

At 12 h post inoculation (h.p.i) with Cry toxins alone, and when combined with spores, ROS production increased by 1.5-fold and MDA levels (i.e., lipid peroxidation) increased by 1.2-fold (Figure 2A,B; *p* < 0.01, q = 3.58; df = 70 and *p* < 0.05, q = 2.76; df = 70, respectively). By 48 h.p.i, ROS levels were similar across all treatments and control, however, MDA continued to increase, rising to ~1.5–1.8-fold compared to the control and spore-treated insects (*p* < 0.05, q = 2.54; df = 74 and *p* < 0.05, q = 2.72; df = 74, respectively; Figure 2A,B). The accumulation of ROS and MDA in toxin and toxin/spore-treated larvae coincided with marked increases in nonspecific esterase activities, i.e., detoxification (Figure 2C). At 12 h.p.i., esterase detected in the midgut of CPB larvae challenged with Bt spores, Cry toxins, or a combination of the two, were significantly higher than the control activity (*p* < 0.05, q = 2.89; df = 79; *p* < 0.05, q = 2.93, df = 79; *p* < 0.05, q = 2.41, df = 79, respectively). Enzyme activity remained 1.5-fold higher in toxin-treated insects (Cry toxins alone and in combination with spores) at 48 h.p.i. (*p* < 0.05, q = 2.80, df = 70; *p* < 0.01, q = 3.22, df = 70, respectively). Enzymatic activity of the antioxidant glutathione-S-transferase (GST) was similar between control and treatments at 12 h.p.i. (<3 Units; Figure 2D), however by 48 h.p.i, GTS activity doubled in the insects exposed to single treatments (spores, 5.9 Units; Cry toxins, 7.1 Units), and tripled to 9.4 Units for the combined dose of Cry toxin/spores (*p* < 0.0001, q = 4.79, df = 76; *p* < 0.0001, q = 6.51, df = 76; *p* < 0.0001, q = 9.65, df = 76, respectively).

**Figure 3:**
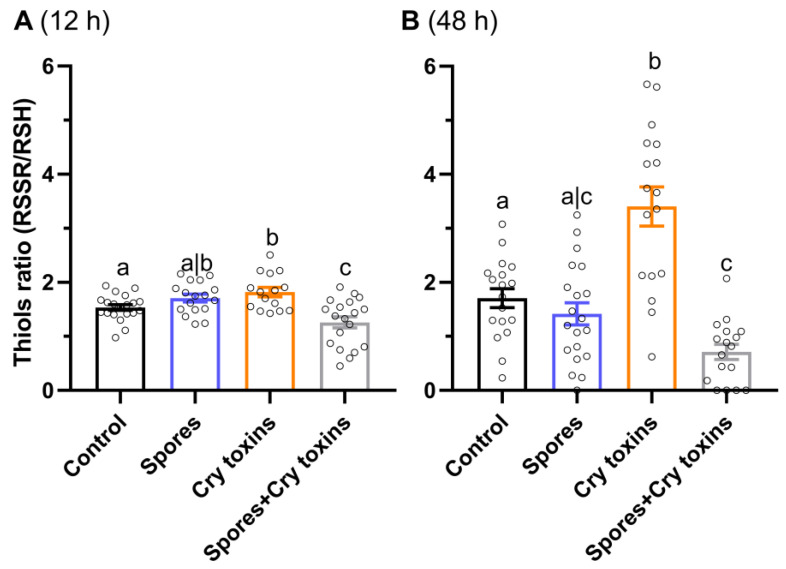
Ratio of oxidized versus reduced thiols (RSSR/RSH) in the midgut of Colorado potato beetle treated with *Bacillus thuringiensis* spores and Cry3A toxins. Measures were taken at 12 (**A**) and 48 (**B**) hours post inoculation. The negative control consisted of administering PBS alone (no spores or toxins). Data represent mean ± SE. Unshared letters represent significant differences (*p* < 0.05) determined via ANOVA (with Dunnett’s multiple comparisons).

The antioxidant ratio of oxidized versus reduced thiol concentrations (RSSR/RSH) in the midgut of larvae dosed with Cry toxins rose by 1.2-fold at 12 h.p.i (*p* < 0.05, q = 2.48, df = 67) and by 2-fold at 48 h.p.i. (*p* < 0.0001, q = 5.06, df = 68) compared to the control group (Figure 3A,B). Interestingly, the combined treatment of Cry toxins and Bt spores decreased the ratio of thiols at both time points, 1.2-fold (*p* < 0.05, q = 2.56, df = 67) and 2.5-fold (*p* < 0.05, q = 2.95, df = 67), when compared to the control. The ratio of thiols in the Cry toxin-treated insects was higher than the Cry toxin/spores combined treatment by 1.5-fold at 12 h.p.i. and 5-fold at 48 h.p.i. (Figure 3A,B). 

### 2.3. Expression of Immunity/Detoxification-Related Genes in the Midgut of CPB Larvae Post Bt Treatment

**Figure 4:**
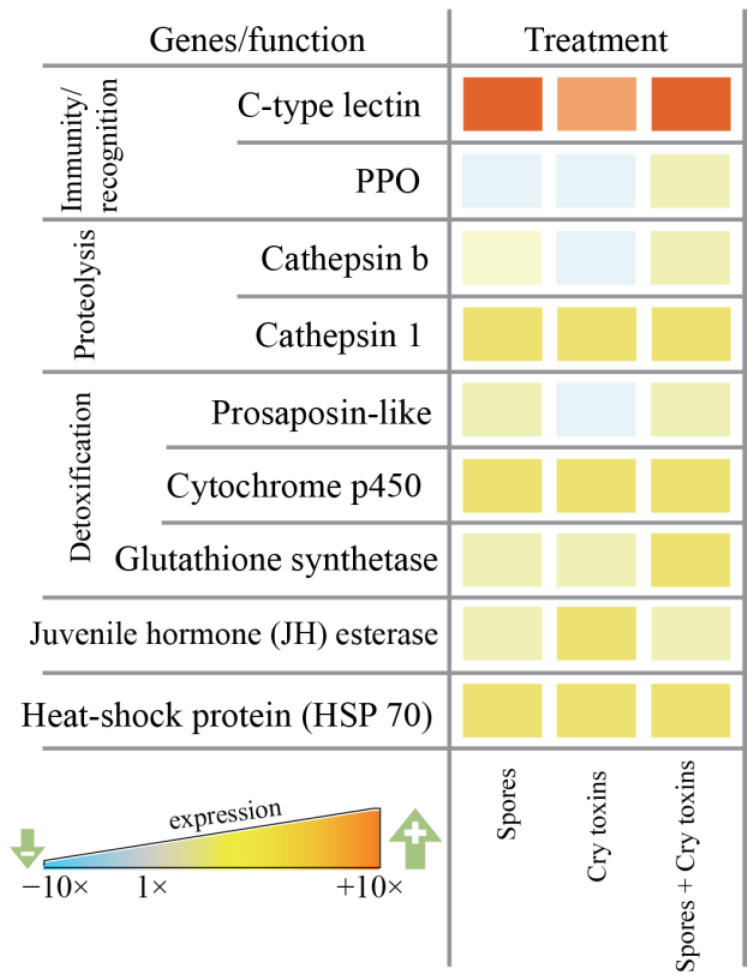
Candidate gene expression in the midgut of Colorado potato beetle larvae exposed orally to *Bacillus thuringiensis* spores and Cry3A toxins. mRNAs were extracted at 48 h post inoculation. Data represent fold change (ΔΔCt value of three independent blocks is reported) relative to the control (PBS) treatment. C-type lectin, prophenoloxidase (PPO), cathepsins b and 1, proactivator polypeptide prosaposin-like, cytochrome p450, glutathione synthetase, juvenile hormone esterase, heat-shock protein (HSP 70).

Differential levels of candidate gene expression (i.e., mRNA) were observed for anti-infective, detoxification and antioxidant factors in the larval midgut 48 h.p.i. with Bt spores, Cry toxins and a combined dose of spores/Cry toxins. Immune-related prophenoloxidase (PPO) remained mostly unchanged, whereas the pathogen recognition (galactose-specific) C-type lectin was upregulated up to 12-fold when compared to the control (Figure 4). Both cathepsins (b and 1) displayed increased expression between 1.5 and 3-fold—these proteases contribute to lysosomal destruction of endogenic and exogenic molecules. Detoxification and stress response factors were also upregulated: 1.5 to 2-fold for proactivator polypeptide prosaposin-like protein, 2 to 3-fold for cytochrome p450 monooxygenase and 1.5 to 2-fold for glutathione synthetase, 1.5 to 3-fold for HSP 70 and juvenile hormone esterase (Figure 4, Supplementary Material Figure S1). 

### 2.4. Microbiota of CPB Larvae Post Bt Treatments

**Figure 5:**
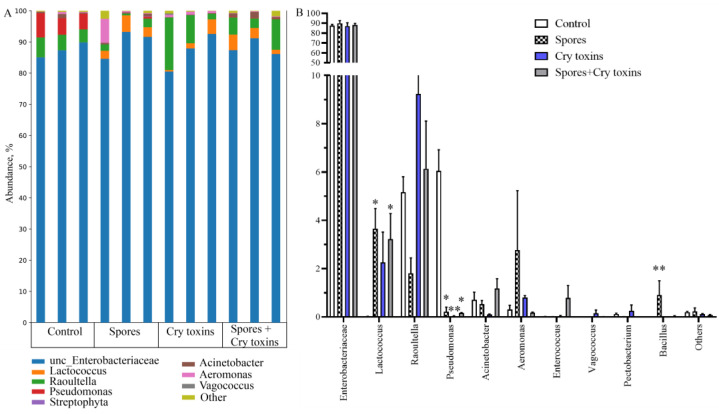
Bacterial microbiome (16s rRNA) profiles in the midgut of Colorado potato beetle larvae treated with *Bacillus thuringiensis* spores and Cry3A toxins. DNA was extracted 48 h post inoculation. (**A**) Bacterial abundance with each biological replicate (*n* = 3 per treatment) displayed. (**B**) Genus-level comparisons. Data represent Mean ± SE (* *p* < 0.05; ** *p* < 0.01compared with the control within the same genus).

Taxonomic classification of bacteria in the midgut of CPB larvae (based on 16S rRNA gene sequencing) revealed communities that were dominated by only a few taxa, with >99% represented by eight genera from four orders (average relative abundances were calculated across all untreated larvae): Enterobacteriales (92.7 ± 0.7%), Pseudomonadales (6.7 ± 0.8), Aeromonadales (0.3 ± 0.05%) and Lactobacillales (0.07 ± 0.03%; Figure 5A). 

Oral inoculation with Bt spores and Cry toxins did not appear to coincide with gross dysbiosis, e.g., enterobacteriaceae in the midgut at 48 h.p.i. Figure 5 represented ~87% in control larvae, 89% in the spore treatment, 87% in the Cry toxin treatment and 88% in the combined spore/toxin treatment. Some fluctuations in bacterial taxa were detected for *Lactococcus*, *Raoultella* and *Pseudomonas*. *Lactococcus* presence was increased in larvae inoculated with Bt spores (*p* < 0.05) and the combined spores/toxins dose (*p* < 0.05). Generally, treated insects displayed lower levels of *Pseudomonas* when compared directly to control larvae (Figure 5B). Data demonstrated that Bt bacteria started to replicate in the midgut of infected insects, but abundance was low at ~1% (variant Spores) and ~0.03% (variant Spores + Cry toxins) (Figure 5B). Richness and diversity indices of midgut bacteria did not alter substantially (Supplementary Material Figure S2). 

**Figure 6:**
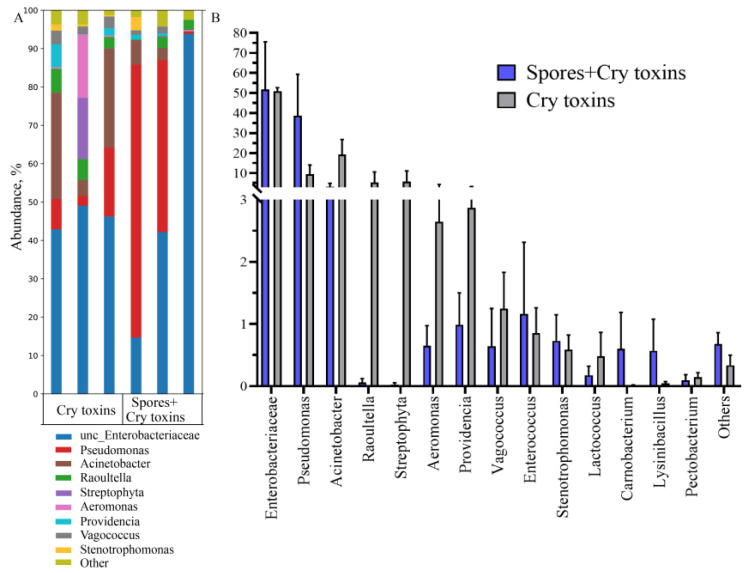
Bacterial microbiome (16s rRNA) profiles in the midgut of beetle cadavers treated with *Bacillus thuringiensis* spores and Cry3A toxins. Bacteria were classified according to genus (**A**) and abundances are expressed with respect to community composition (**B**). DNA was extracted post-mortem. Data presented as Mean ± SE (*n* = 3 per treatment).

Taxonomic classification of bacteria taken from larval cadavers (48 h.p.i.) with Cry toxin alone or combined with spores housed 99.5% bacterial communities across 14 genera, with *Enterobacteriaceae*, *Pseudomonas*, and *Acinetobacter* the dominant taxa (Figure 6A). Enterobacteriaceae represented 47–50% of bacteria in cadavers and were 1.8 to 2-fold fewer when compared to the midguts of insects that survived Cry toxin treatments. Significant multiplication of *Pseudomonas* (10–40%) and Acinetobacter (3–20%) were observed in intoxicated cadavers. (Figure 6B). Again, the richness and diversity indices of midgut bacterial communities remained unchanged (Supplementary Material Figure S3). 

## 3. Discussion

The present study shows that Bt spores synergistically enhance the insecticidal potency of Cry3A toxins in CPB larvae. When combined, spores and Cry toxins lead to the degeneration of midgut physiology—most likely due to metabolic derangement from the accumulation of noxious reactive oxygen and nitrogen radicals. During the early stage of pathogenesis (12 h.p.i.), the sublethal dose of Cry toxins compromises the lipid peroxidation of the midgut epithelium (evident from MDA levels). The insect goes some way to counteract the damage by activating enzymatic and chemical detoxification machinery and the upregulation of the cell stress response. However, Bt spores combined with Cry toxins cause these controls to fail. At the later stage of infection, if insects are still living, the detoxification system activated under Bt spores, Cry toxins, and their combined doses, mostly mitigate the toxicosis at 48 h.p.i. Interestingly, Bt spores and/or Cry toxins do not alter the midgut bacterial composition much after 48 h in living insects, but those that die at the same time-point do show altered abundances of various genera and clear evidence of Bt replication—presumably in preparation of sporulation. 

It is well known that spores can increase the potency of Bt Cry toxins in some insects. Synergistic interactions of spores and Cry toxins depend on the characteristics of Bt-insect parasite systems (e.g., bacteria, dose of inoculum and age of larvae). Mostly, synergistic interactions have been detected in lepidopteran species where spores increased the mortality of wax moths (3–15 fold) [38], Indianmeal moths (25–44 fold) [40] and diamondback moths (5–100 fold) [39]. Our study is the first to characterise the synergy of Bt spores on the insecticidal activity of Cry3A toxins in the Colorado potato beetle (order Coleoptera). Spores, together with Cry toxins, enhance mortality of the CPB larvae by 2- to 3-fold. This synergistic activity may be caused by a complex of additional virulence factors that bacterial spores bring to pathological processes. Particularly, spores of Bt can carry Cry toxins [44] and metalloprotease (InhA1) on the cellular surface [45]. InhA1 is a potent virulence factor as it directly enhances the activity of Cry toxins, destroying tissues and degrading the antimicrobial proteins of wax moth larvae [46,47,48]. The Vip toxins secreted by Bt during vegetative growth entering into the brush border membrane (of the midgut) after enzymatic activation caused high levels of toxicity in *Holotrichia parallela* (Coleoptera: Scarabaeidae) [49]. Bt bacterial chitin-binding protein with chitinase activity detected in wax moth infected with Bt are able to take part in the development of gut toxicosis in the wax moth, due to the destruction of the peritrophic membrane and improvements in the passage of the Cry toxins to the epithelial cells [50]. 

The insect gut is the first and most important barrier against oral bacterial (Bt) infection [21,51]. We found that exposure to Cry toxins leads to dysregulated lipid peroxidation on the surface of midgut epithelial cells of CPB larvae and increased ROS production. Redox imbalance in the gut tissues is a pathophysiological symptom of bacterial Bt infection [52]. Development/evolution of insect resistance to Bt across generations coincides with a more robust antioxidant system [21]. Data collected in *P. xylostella* also demonstrated that Bt led to the upregulation of ROS levels in the gut lumen; however, ROS balance is also important for insect defence against bacteria [53]. We found that the midgut redox balance of CPB larvae treated with Cry toxins alone in sublethal doses is neutralised successfully with nonenzymic thiol antioxidants. This led to the normalization of ROS generation level to control at 48 h post-treatment. Thiols are critical in redox balance control because they are one of the main insect antioxidants that control ROS generation during infections and immune response [33,52]. However, spores added to Cry toxins resulted in a critical redox imbalance in the midgut from 12 to 48 h post-treatment. Insects demonstrated high level of ROS production and lipid peroxidation simultaneously with low thiol antioxidant activity. Collectively, these results are evidence of “oxidative stress” (the shift in the balance between oxidants and antioxidants) in the midgut of CPB larvae. Maintenance of optimal redox balance in midgut of CPB larvae infected with Bt modulated by antioxidant system is critical to both insect antibacterial defence reactions and the protection of its own cells from the ROS. In the development of the pathogenesis in the presence of Bt spores and Cry toxins, the imbalance of the ROS-antioxidant system persists, which is likely facilitating the synergistic action of bacteria cells/spores and the Cry toxins.

The detoxification system is activated in response to the endogenous and exogenous toxins/by-products in insects. Nonspecific esterases and GST play an active role in toxin inactivation during fungal *M. robertsii* and bacterial *B. thuringiensis* infections in CPB larvae in fat body and haemolymph [54]. The detoxification system also maintains the development of immune reactions related to ROS production (encapsulation, melanisation) [33]. During the development of Bt infection in the midgut of CPB larvae, induction of some detoxification system components have been determined, such as esterases, GST, cytochrome p450 monooxygenase, glutathione synthetase and saposins (prosaposin-like precursors) and cathepsin-proteases that take part in the lysosomal destruction of endogenic and exogenic molecules [23]. Some studies suggest that esterase plays an important role in the midgut defense of *G. mellonella* and *H. armigera* larvae against *Bt* [24,55]. Interestingly, separate treatment with Bt spores resulted in activation of the esterases (12 h) and GST (48 h) post treatment. Probably virulence factors introduced by spores can also induce a detoxification system response, which indicates their independent toxic activity. Activation of enzymes is more pronounced when CPB larvae are exposed to Cry toxins alone, and when Cry toxins are combined with spores, than when exposed to spores alone. 

Insects demonstrate complex local and systemic innate immune responses to Bt infection. The cellular and humoral reactions are triggered systemically in the hemolymph [56,57,58], as well as locally via antimicrobial peptides production detoxification and regeneration in the midgut [21,53,59,60,61]. However, the local immune reactions at the primary source of bacterial penetration (midgut) are vital in order to circumscribe infection and avoid septicaemia. This is supported by major trends in the formation of insect resistance to Bt through receptor mutations to the Cry toxins on the surface of epithelial cells of midgut [62], and enhanced midgut immunity [21]. Herein, the inoculation of CPB larvae with Bt led to the activation of genes responsible for immunity, detoxification and stress mitigation when exposed to Cry toxins, spores and their mixture. Interestingly, all treatments resulted in elevated levels of galactose-specific C-type lectin that are responsible for recognizing antigens of various pathogens [63] and cathepsins linked to Toll signalling cascades [64,65]. Cells of the beetle midgut are recognising and triggering immune responses to both spores that carry certain antigens (PAMPs) and Cry toxins through damage-associated mechanisms (DAMPs) [22]. Interestingly, the enhanced expression of the juvenile hormone esterase gene destroys the juvenile hormone, and is linked to metamorphosis and immune-modulation during Bt infection [66,67]. Elevated expression of the gene encoding the stress-associated heat shock protein 70 (HSP 70) was also observed in toxin/spore challenged CPB larvae. 

Bt bacteria themselves can cause septicaemia, as well as displacement of bacteria from native midgut microbiota, which can also pose a risk. There are links between the bacterial microbiota and Bt virulence [35,68]. The present study shows all Bt treatment of CPB led to some changes in midgut microbiota of CPB larvae, e.g., *Lactococcus* and *Raoultella* presence. In a similar study, dramatic shifts in the midgut bacterial community under Bt treatment, which was mainly related with depletion of endosymbiotic bacteria *Spiroplasma leptinotarsae*, under bacterial toxicosis was observed [69]. In our study, the midgut of the Bt spore/toxin-inoculated CPB larvae was not conducive to *Pseudomonas* growth. The exact mechanisms altering the gut environment have not been identified but may include the secretion of AMPs, and/or the removal of antagonistic microbes [70]. We found the CBP midgut immunity was enhanced post Bt treatment and this would have significant benefits by reducing the danger of septicaemia and secondary infections. Spontaneous bacteriosis in insects has been considered an additional mechanism by which Bt may kill and colonize their hosts [6,71]. Thus, Pseudomonas may be as an additional factor enhancing the pathogenesis of Bt because dysregulated gut environments in insects under Bt treatment could make it possible to convert some symbiotic mutualistic bacteria into opportunistic pathogens, enhancing their abundance in cadavers [36]. However, the relationships of bacterial consortia in cadavers are complex and require further study. 

## 4. Conclusions

CPB larvae demonstrate complex local defence responses in the midgut when infected with Bt, their spores and/or Cry3A toxins. Midgut antioxidants, detoxification enzymes and immune factors are used to counter Bt toxin-induced pathogenesis. Spores of Bt synergistically enhance the toxicity of Cry toxins—leading to higher rates of mortality and speed of kill. ROS dysregulation and an overloaded antioxidant system appear to be key features of Bt pathophysiology in CPB. Additional virulence factors involved in Bt pathogenesis, which offers scope for further study, are likely found in both spores and vegetative cells that assist Cry toxins. Using Bt crystal endotoxins with spores together represents a promising avenue for pest management programs. 

## 5. Materials and Methods

### 5.1. Insects and Bacteria 

CPB larvae were collected from the potato *Solanum tuberosum* in the Novosibirsk region (55.0321663022145 N, 82.9903430545771 E), free of insecticides. Larvae were maintained under 12/12 h light/dark cycle at 25 °C. Larvae were kept in plastic containers (300-mL) with 10 insects per container, and were fed with potato leaves placed in 1.5 mL tubes with water. Potato shoots were changed daily. Between 4 and 6 h after moulting at the fourth instar, larvae were used for experiments. 

The bacterium *Bacillus thuringiensis* ssp. morrisoni var. thuringiensis strain Btm19 from Novosibirsk State Agrarian University collection was used to infect the CPB larvae. Bacteria were cultured on plates of Luria–Bertani medium (LB, 1% tryptone, 0.5% yeast extract, 1% NaCl in *w*/*v*, pH 7.0) at 30 °C until complete autolysis. Spores and crystals of the bacteria were resuspended in 10 mM phosphate buffer containing 150 mM NaCl, pH 7.2 (PBS) and washed twice with saline solution (NaCl 0.9% *w*/*v*) at 6000× *g* for 10 min at 4 °C. Collected spore-crystal mixtures (1:1) were resuspended in PBS and separated by sucrose density gradients [72]. Crystal endotoxin (square-shaped) of Bt ssp. morrisoni var. *thuringiensis* contain Cry3A toxin, 65 kDa in size. For insect inoculation, native crystal endotoxins were used.

Oral inoculation was used for CPB larvae treatment with Bt spores, crystals or its mixture by force-feeding with a hypodermic needle (30G) and syringe pump (KDS 100, KD Scientific). Each larva was inoculated with 10 μL suspension of bacterial spores (5 × 10^8^ in PBS), crystals (5 × 10^8^ in PBS) and bacterial spore–crystal mix (*n* = 1000 per treatment). The number of spores and crystals were counted using a haemocytometer. The negative control insect group were force-fed 10 µL PBS. Larval mortality was recorded daily over 8 days (*n* = 100 per treatment). Inoculated fourth instar insects were collected 12 h post-exposure to Bt in order to: (1) determine the activity of enzymes (esterase, GST) in the midgut (individual midgut in one sample, *n* = 20 per treatment); (2) measure redox balance (lipid peroxidation, thiols) in the midgut (5 midgut pooled in one sample, *n* = 20 per treatment for lipid peroxidation; 3 midgut pooled in one sample, *n* = 20 per treatment for thiols) and 48 h post-exposure to Bt to: (1) determine the activity of enzymes (esterase, GST) in the midgut (individual midgut in one sample, *n* = 20 per treatment); (2) measure redox balance (lipid peroxidation, thiols) in the midgut (5 midgut pooled in one sample, *n* = 20 per treatment for lipid peroxidation; 3 midgut pooled in one sample, *n* = 20 per treatment for thiols); (3) quantify gene expression in the midgut (3 midgut pooled in one sample, *n* = 5 per treatment) and (4) determine bacterial content of the midgut (5 midgut pooled in one sample, *n* = 3 per treatment) in control, Bt spores, crystals and bacterial spore–crystal mix treatments. Bacterial content in cadavers (5 larvae pooled in one sample, *n* = 3 per treatment) was analysed for variants crystals and bacterial spore–crystal mix treatments (48 h post-exposure).

### 5.2. Enzymatic Activity and Redox Balance in Midgut of CPB Larvae

Midgut dissection from surface-sterilized larvae (esterase, GST, ROS generation, one larva per sample; lipid peroxidation, five larvae per sample) was carried out in PBS. The dissected midgut was sonicated in 100 µL of PBS. The homogenates were centrifuged for 15 min, 10,000× *g* at 4 °C. The supernatant was used for the analysis of enzyme activity and redox balance.

Nonspecific esterase activity was estimated using p-nitrophenyl acetate hydrolysis rate following Prabhakaran et al. [73]. Samples (5 µL) were incubated for 10 min with 200 µL p-nitrophenyl acetate at 28 °C, then, the absorbance was measured at 410 nm. The activity of GST against 2-nitro-5-thiobenzoic acid (DNTB) was estimated by the method of Habig [74]. Incubation of a 10 µL sample was performed with 1 mM glutathione and 1 mM DNTB at 25 °C for 10 min. The concentration of 5-(2,4-dinitrophenyl)-glutathione was recorded at 340 nm. Esterase and GST activities were converted to units of transmission density (∆α) of the incubation mixture per min and 1 mg of protein. 

DCFH_2_ (2′,7′-dichlorodihydrofluorescein) was used in vitro for measuring total ROS/RNS free radical activity from the homogenates of midguts. Five µL of sample was added each well with 200 µL of the DCFH_2_ solution (10 µM in PBS) and the oxidation reaction was incubated at 37 °C for 30 min. Samples were measured fluorometrically (Ex/Em = 485/530 nm). ROS generation is presented as fluorescence ((Ft_30_-Ft_0_), where Ft_30_ = fluorescence at time 30 min and Ft_0_ = fluorescence at time 0 min) per mg protein [75].

The process of lipid peroxidation results in the formation of malondialdehyde (MDA). This is a later product in the sequence of lipid peroxidation reactions [76]. The thiobarbituric acid (TBA) assay was used to assess MDA concentration, with some modifications, as described in Bar–Or et al. [77]. A total of 20 µL of 20% TBA was mixed with 40 µL of the sample, after which the mixture was centrifuged at 10,000× *g* for 10 min at 4 °C. Supernatant (50 µL) was mixed with 150 µL of 0.8% TBA, and incubated at 100 °C for 60 min. The MDA–TBA adduct was quantified fluorometrically (Ex/Em = 532/553 nm). The MDA concentration is presented as nmoles of MDA per mg protein using t1,1,3,3-tetramethoxypropane as a standard. The concentration of protein in the homogenates was determined by the Bradford method (Bradford, 1976) using bovine serum albumin (BSA) for the calibration curve.

To determine the thiols RSSR/RSH ratio, a method based on reduced thiols (RSH) oxidation by DTNB was used [33,78]. Prior to spectrophotometric analysis, oxidized thiols (RSSR) were incubated for 20 min by 1M hydrochloric acid to form RSH; the pH of the mixture was then neutralized (pH 7) with sodium hydroxide. Sample (50 µL) was mixed with 500 µL of 0.1% 5,5-dithiobis-(2-nitrobenzoic acid) solution in PBS, and incubated for 10 min at 37 °C. Cysteine was used to prepare a calibration curve. The absorbances were measured at 412 nm. The results are presented as the ratio of RSSR to RSH.

### 5.3. QRT–PCR Analysis of Insect Immunity and Stress-Related Gene Expression in the Midgut 

Nine genes previously attributed to anti-infective, stress, detoxification and antioxidant responses in CPB were investigated: galactose-specific C-type lectin, prophenoloxidase (PPO) [22], cathepsin b and cathepsin 1 proteases (involved in lysosomal destruction of endogenic and exogenic molecules [23]), detoxification proteins and enzymes-proactivator polypeptide prosaposin-like protein [23], cytochrome p450 monooxygenase and glutathione synthetase, genes encoding the stress-associated protein chaperone, heat shock protein 70 (HSP 70) and juvenile hormone metabolism [79].

Midgut tissues were dissected from surface-sterilized larvae (three larvae per sample) and stored in RNA-later (Ambion) before QRT-PCR analysis of insect gene expression. Samples were freeze-dried and crushed in liquid nitrogen. Total RNA was isolated using TRIzol^®^ Reagent (Invitrogen) according to the manufacturer’s recommendations. RNA concentrations were determined by nanophotometer (Implen), and 2 μg of RNA was treated with DNAase I (Promega), at 37 °C for 30 min. Complementary DNA synthesis was performed with 1 μg RNA using the qScript™ cDNA SuperMix (Quanta Bioscience). 

cDNA quantity was checked using the reference gene of 1/50 dilution of each sample measured against a standard curve, and sufficient cDNA of similar concentration for each sample diluted to amplify all genes. Samples were quality checked for consistency between values for the two reference genes used: *Rp4* (KC190033.1) and *Rp18* (KC190034.1). Expression was measured among normalised samples using the CFX96 Real-Time PCR detection system (Biorad). Primers were designed from published *Leptinotarsa decemlineata* genome/sequences (NCBI), transcriptome (RNAseq) (SRX017239) and are given in Supplementary Material Table S1. Other primers were designed using Primer3 [80] to amplify at 60 °C with an amplicon size of 80–200 bp, rechecked for potential dimer formation with Oligo 6 (Molecular Biology Insights, Inc., Colorado Springs, CO, USA), and for amplicon secondary structure using the Mfold server [81]. Primers were optimised by checking products for a clean single peak by HRM (high resolution melt curve)) analysis and by titrating concentration for optimal efficiency using a serial dilution of mixed cDNA. 

A mix of 5 μL SYBR Green Fastmix (Biolabmix, Novosibirsk, Russia) and 1 μL of equimolar primer mix was added to 4 μL cDNA for each 10 μL PCR reaction. Cycling conditions were 95 °C for 5 min followed by 39 cycles of: 95 °C 15 s, annealing 15 s, 72 °C 30 s. HRM analysis was performed at the end of each run. All reactions were performed in triplicate, and optimal threshold values and reaction efficiencies calculated from 7-point serial dilutions of mixed cDNA from infected insects. Fold change values were calculated using the ΔΔCt method: for each locus, the ΔΔCt for sample was determined by subtracting the measured Ct value from the Ct value of each reference or ‘housekeeping’ gene. ΔΔCts were then converted to relative copy numbers with the formula 2 ^∆∆∆Ct^. Fold changes were also calculated using reaction efficiencies using the *Pffafl* equation [82]. Values showed similar trends for both reference genes and for each method of calculation: ΔΔCt values for *Rp4* are shown.

### 5.4. 16S (V3/V4) rDNA Bacterial Diversity Analysis of Midgut and Cadaver Community of CPB

The bacterial community in the midgut of CPB larvae inoculated with Bt spores, Cry toxins, mixture of spores with Cry toxins (48 h post inoculation) was analysed by 16S rDNA metagenomics sequencing. Midguts with intact contents were dissected from surface-sterilized larvae (five larvae per sample) or larval cadavers (five larvae per sample) and frozen in liquid nitrogen. DNA was isolated using the DNeasy PowerSoil Kit (Qiagen, Hilden, Germany). The homogenization was made using TissueLyser II (Qiagen) 10 min at 30 Hz. The V3-V4 region of the 16S rRNA genes was amplified with the primer pair 343F and 806R [83]. The 16S libraries were sequenced with 2 × 300 bp paired-ends reads on MiSeq (Illumina, San Diego, CA, USA) in SB RAS Genomics Core Facility (ICBFM SB RAS, Novosibirsk, Russia). The MiSeq data were deposited in GenBank under the study accession number PRJNA747108.

Raw sequences were analysed using the UPARSE pipeline [84] and Usearch v11.0.667. The UPARSE pipeline included merging of paired reads; read quality filtering; length trimming; merging of identical reads (dereplication); discarding single reads; removing chimeras and OTU clustering using the UPARSE algorithm [85]. The OTU sequences were assigned taxonomy using the SINTAX [86] and 16S RDP training set v16 as a reference [87]. Alpha diversity metrics were calculated in Usearch.

### 5.5. Data Analyses

Data are presented as the mean ± standard error. Data were checked for Gaussian distribution using the D’Agostino–Pearson omnibus test, and if non-normal, a conservative non-parametric analysis was applied. We used parametric analysis (ordinary one-way ANOVA with Dunnett’s multiple comparisons test) to analyse the enzymatic activity and redox data. Nonparametric one-way ANOVA (Kruskal–Wallis) with Dunn’s multiple comparisons test was used to determine the differences between insect immunity and stress related gene expression (QRT-PCR analysis). Midgut and cadaver microbiota of CPB larvae post Bt treatments were analysed with nonparametric one-way ANOVA with Dunn’s multiple comparisons test (for abundance) and nonparametric t-test (Mann–Whitney test) for richness and diversity. Survival was calculated using the product limit (Kaplan–Meier) method. Analyses for additive, antagonistic and synergistic interactions were based on a binomial test, which involved comparing the expected and observed mortalities [88]. Richness and Cox’s proportional hazards survival regression (Log-rank (Mantel–Cox) test) was used to quantify the differences in mortality rates. Data analyses were performed using (GraphPad Prism v8.0 (GraphPad Software, San Diego, CA, USA). 

## Figures and Tables

**Table 1:** Survival analyses of CPB larvae exposed to Bt toxins and spores alone, or in combination. Outputs from pairwise comparisons using the Log-rank (Mantel–Cox) test and Mantel–Haenszel hazard ratio (HR). Significant differences (*p* < 0.05) are in bold.

	Spores	Cry3A Toxins	Spores + Cry3A Toxins
Control	*X*^2^ (1) = 0.988, *p* = 0.3201	*X*^2^ (1) = 11.64, ***p* = 0.0006** HR = 3.9 (95% CI, 1.8–8.8)	*X*^2^ (1) = 46.75, ***p* < 0.0001** HR = 6.3 (95% CI, 3.7–10.6)
Spores	-	*X*^2^ (1) = 6.224, ***p* = 0.0126** HR = 2.6 (95% CI, 1.2–5.5)	*X*^2^ (1) = 36.05, ***p* < 0.0001** HR = 4.9 (95% CI, 2.9–8.2)
Cry3A toxins	-	-	*X*^2^ (1) = 15.92, ***p* < 0.0001** HR = 2.6 (95% CI, 1.7–4.1)

## Data Availability

The data presented in this study are available upon request to the corresponding author.

## Supplementary Materials

The following are available online at https://www.mdpi.com/article/10.3390/toxins13110746/s1, Figure S1. Candidate gene expression in the midgut of Colorado potato beetle larvae exposed orally to Bacillus thuringiensis spores and Cry3A toxins, Figure S2: Richness and diversity indices for bacteria in midgut of Colorado potato beetle larvae at 48 h post treatments with *Bacillus thuringiensis*, Figure S3: Richness and diversity indices for bacteria in cadavers at 48 h post treatment of Colorado potato beetle larvae with *Bacillus thuringiensis*, Table S1: Loci used for expression analysis.
