# Supplementary material for: Bacillus thuringiensis Spores and Cry3A Toxins Act Synergistically to Expedite Colorado Potato Beetle Mortality
Source: Toxins (Basel). 2021 Oct 21;13(11):746. doi: 10.3390/toxins13110746 (PMC8624055; doi:10.3390/toxins13110746)
Supplement: Supplementary file 1 [file toxins-13-00746-s001.zip › toxins-1394438-supplementary/Supplementary material - figures.pdf]

Supplementary Materials: *Bacillus thuringiensis* Spores and Cry3A Toxins Act Synergistically to Expedite Colorado Potato Beetle Mortality

Ivan M. Dubovskiy, Ekaterina V. Grizanova, Daria Tereshchenko, Tatiana I. Krytsyna, Tatyana Alikina, Galina Kalmykova, Marsel Kabilov and Christopher J. Coates

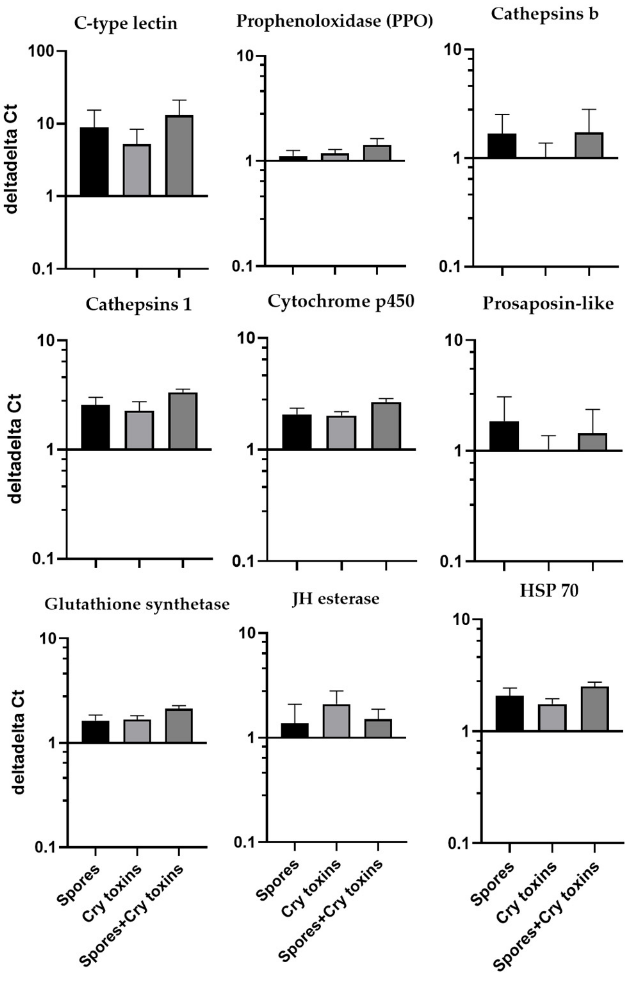

Figure S1. Candidate gene expression in the midgut of Colorado potato beetle larvae exposed orally to *Bacillus thuringiensis* spores and Cry3A toxins. mRNAs were extracted at 48 h post inoculation. Data represent fold change (ΔΔCt value of three independent blocks is reported) relative to the control (PBS) treatment. C-type lectin, prophenoloxidase (PPO), cathepsins b and 1, proactivator polypeptide prosaposin-like, cytochrome p450, glutathione synthetase, juvenile hormone (JH) esterase, heat-shock protein (HSP 70).

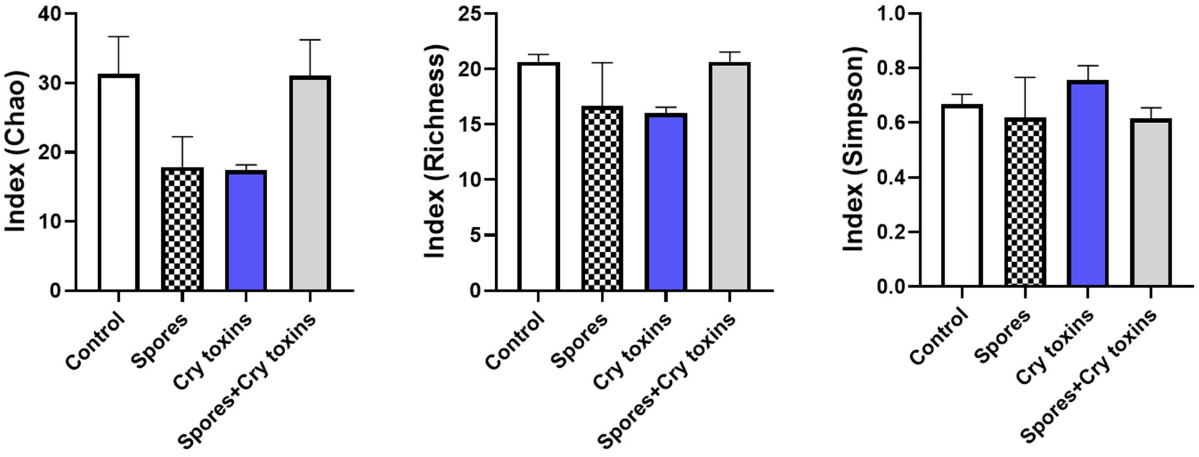

Figure S2. Richness and diversity indices for bacteria in midgut of Colorado potato beetle larvae at 48 h post treatments with *Bacillus thuringiensis* spores, Cry3A toxins, mixture spores with Cry3A toxins and PBS (Control). Chao (A) and Richness (B) community quantitative index reflecting richness (i.e., different bacterial phylotypes) in a dataset. Simpson (C) index reflecting diversity of bacterial communities. Data presented as Mean ± SE.

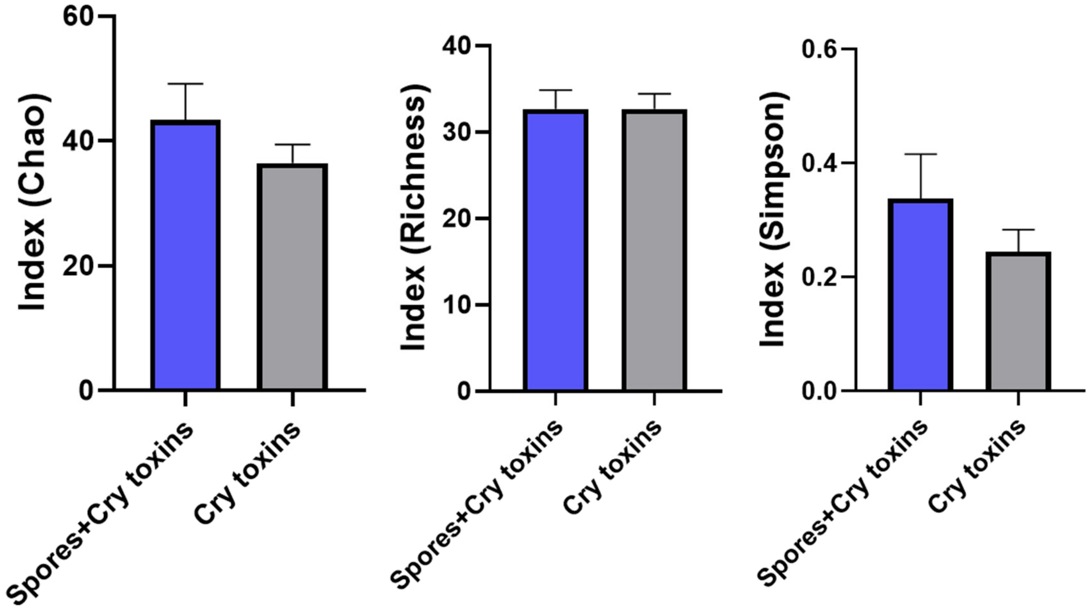

Figure S3. Richness and diversity indices for bacteria in cadavers at 48 h post treatment of Colorado potato beetle larvae with *Bacillus thuringiensis* spores, Cry3A toxins, mixture spores with Cry3A toxins and PBS (Control). Chao (A) and Richness (B) community quantitative index reflecting richness (i.e., different bacterial phylotypes) in a dataset. Simpson (C) index reflecting diversity of bacterial communities. Data presented as Mean ± SE.
